# Longterm Reversal of Severe Visual Loss by Mitochondrial Gene Transfer in a Mouse Model of Leber Hereditary Optic Neuropathy

**DOI:** 10.1038/s41598-018-23836-y

**Published:** 2018-04-03

**Authors:** Hong Yu, Vittorio Porciatti, Alfred Lewin, William Hauswirth, John Guy

**Affiliations:** 1 Bascom Palmer Eye Institute, University of Miami, Miller School of Medicine, Miami, FL United States; 2 Department of Molecular Genetics and Microbiology, University of Florida College of Medicine, Gainesville, FL United States; 3 Department of Ophthalmology, University of Florida College of Medicine, Gainesville, FL United States

## Abstract

In many human disorders mitochondrial dysfunction is central to degeneration of retinal ganglion cells. As these cells do not regenerate, vision is irreversibly lost. Here we show reversal of visual dysfunction by a mitochondrially targeted adeno associated virus in transgenic mice harboring a G11778A mutation in the *ND4* subunit of complex I persists longterm and it is associated with reduced loss of RGCs and their axons, improved oxidative phosphorylation, persistence of transferred *ND4* DNA and transcription of *ND4* mRNA.

## Introduction

Visual loss from optic neuropathy is estimated to affect more than 50 million people^1^, and glaucoma is the leading cause. In this disease, once retinal ganglion cells (RGCs) whose axons comprise the optic nerve are gone, visual loss is irreversible^2^. Recent evidence suggests that mitochondrial dysfunction drives RGC loss and that this sequelus can be prevented by modulating mitochondrial function^3^. Leber hereditary optic neuropathy (LHON) is an uncommon cause of optic neuropathy, but it is one of the most common diseases caused by mutations in the mitochondrial genome^4^. Unlike most other hereditary optic neuropathies and glaucoma that are slowly progressive, visual loss in LHON is typically acute in onset, bilateral and severe. Over 95% of all cases are due to pathogenic mutations in one of three mitochondrial genes that encode complex I subunits of the respiratory chain: *ND1G3460A*, *ND4G11778A* or *ND6T14484C*^5^. These mutations impair oxidative phosphorylation and promote death of RGCs. Effective treatments that reverse the visual loss of mitochondrial diseases have yet to be realized^6,7^. Here we utilize a mitochondrially targeted adeno associated viral vector (MTS-AAV) to deliver the wild-type human *ND4* gene into RGC mitochondria^8^ of mitomice that transmit the human *ND4G11778A* gene as a mitochondrial episome^9^ and demonstrate the long term persistence of reversal of the adverse effects of the mutant gene on the visual system.

## Results

### Wild-type *ND4* mediates prompt and persistent reversal of visual function loss in mutant *ND4* mitomice

**Figure 1:**
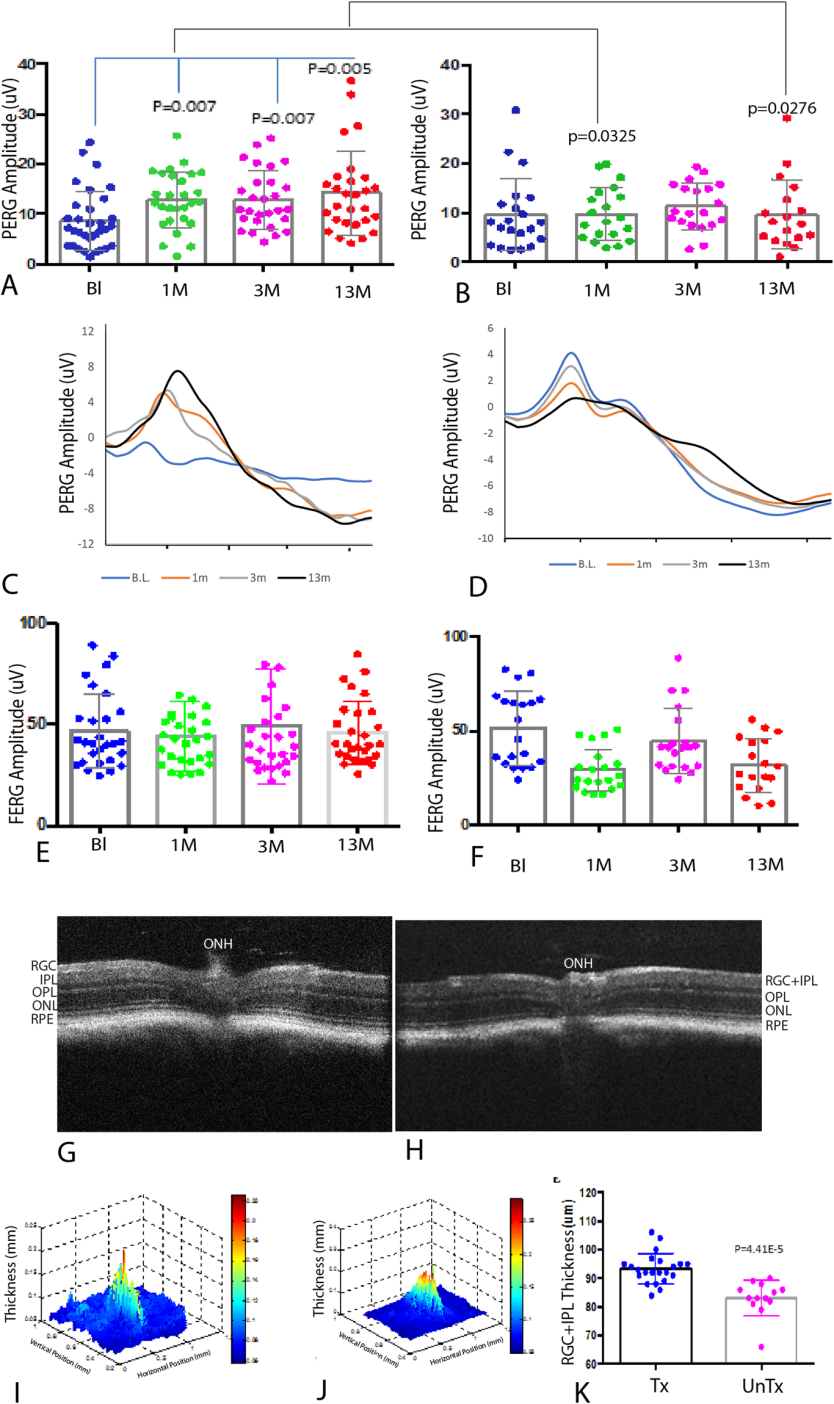
(**A**) Scatterplots of 3-month old transgenic mice with low PERG amplitudes at baseline (Bl) show amplitudes rise 1 (1 M), 3 (3 M), and 13 months (13 M) after intravitreal injection of MTSAAV containing the wild-type human *ND4*(MTSAAV-*hND4*). (**B**) Scatterplots of the littermate controls with low PERG amplitudes at baseline remain low 1, 3, and 13 months after intravitreal vector injection of MTSAAV carrying *mCherry*. (**C**) Representative PERG waveforms of MTSAAV-*hND4* treated mice and untreated mice (**D**) with the x axis in 100-ms increments. Flash ERGs of MTSAAV-*hND4* treated mice (**E**) and MTSAAV-*mCherry* injected controls (**F**) show no differences. (**G**) OCT 2D imaging of an MTSAAV-*hND4* treated mouse eye 12 months postinjection shows the retinal ganglion cell layer plus inner plexiform layer appears thicker than an MTSAAV-*cherry* injected control (**H**). ONH = optic nerve head, RGC = retinal ganglion cell layer, IPL = inner plexiform layer, OPL = outer plexiform layer, ONL = outer nuclear layer, RPE = retinal pigment epithelial layer. (**I**) Representative 3D thickness maps of an MTSAAV-*hND4* treated mouse and (**J**) an MTSAAV-m*Cherry* injected control 12 months after injection. (**K**) Scatterplots show average thickness measurements of the RGC + IPL 12 months after intraocular injections.

To determine whether visual loss of G11778A mitomice is reversible, both eyes of 3 month old G11778A mitomice (n = 14) with low PERG amplitudes were injected with mitochondrially targeted AAV containing the wild-type (WT) human *ND4* (MTSAAV-*hND4*) (Supp. Figure 1). In addition, both eyes of littermates with low PERG amplitudes (n = 10) were injected with mitochondrially targeted AAV carrying the *mCherry* gene in the mitochondrial genetic code (MTS-AAV-m*Cherry*) (Supp. Figure 1, open circles or square). Gene therapy with wild-type *ND4* reversed loss of PERG amplitudes with an increase of 17% at one month post injection (p = 0.007) that improved further to 42% at three months post injection (p = 0.007) (Fig. 1A). In contrast, PERG amplitudes of *mCherry* injected eyes remained low (Fig. 1B). While encouraging, it is unclear whether initial benefits to visual function return would diminish as seen in Leber Congenital Amaurosis (LCA2), a non-mitochondrial disease caused by recessive mutations in the *RPE65* gene, where topographical maps of visual sensitivity in retinal regions treated by AAV delivery of the normal RPE65 cDNA had progressive diminution of the areas of improved vision 4–6 years after treatment^10^.

Next, we tested for treatment persistence. Longer-term follow up of our *hND4* treated mice showed stable visual function with a 46% increase in the PERG amplitude (Fig. 1A) relative to *mCherry* injected controls (Fig. 1B), p = 0.0276, 13 months postinjection. Relative to baseline values obtained prior to *hND4* injections PERG amplitude increased 64%, p = 0.005. Representative waveforms of *hND4* vector treated mutant *ND4* mitomice mice at each time point (Fig. 1C) illustrated that *hND4* vector improved visual function. PERG amplitudes of individual mice improved in most (Supp. Figure 2A–K), but not all eyes (Supp. Figure 2L–O). In contrast, PERG amplitudes of most (Supp. Fig. 3A–E), but not all (Supp. Fig. 3F–I) untreated mice eyes deteriorated (Fig. 1D). Flash ERGs, a measure of global retinal function, showed no changes (Fig. 1E,F) confirming that the reversal of visual dysfunction measured by the PERG was due to effects on RGCs and not photoreceptors^11^.

### Wild-type *ND4* reduces loss of RGCs and optic nerve axons

Next, we used optical coherence tomography (OCT) to look for loss of RGCs in the retinas of live affected mice. Unlike human LHON where OCT can identify changes in thickness of the RGC layer^12^, in the mouse the RGC layer itself is beyond the dependable resolution of the instrument, so here we measured the RGC + inner plexiform (IPL) layers. Representative OCTs of treated (Fig. 1G) and untreated (Fig. 1H) eyes at 12 months of age show a thicker RGC + inner plexiform (IPL) layer in *hND4* vector injected mice. Segmentation revealed that differences in thickness maps of RGC + IPL thickness of *hND4* treated mice (93 ± 5 μm; mean ± SD) (Fig. 1I) and *mcherry* control mice (83 ± 6 μm; mean ± SD) (Fig. 1J) were statistically significant (p = 4.41e^−5^) (Fig. 1K). While this finding may suggest MTSAAV-*hND4* treatment attenuated loss of RGCs, it could also suggest it caused swelling of the RGC + IPL layers.

**Figure 2:**
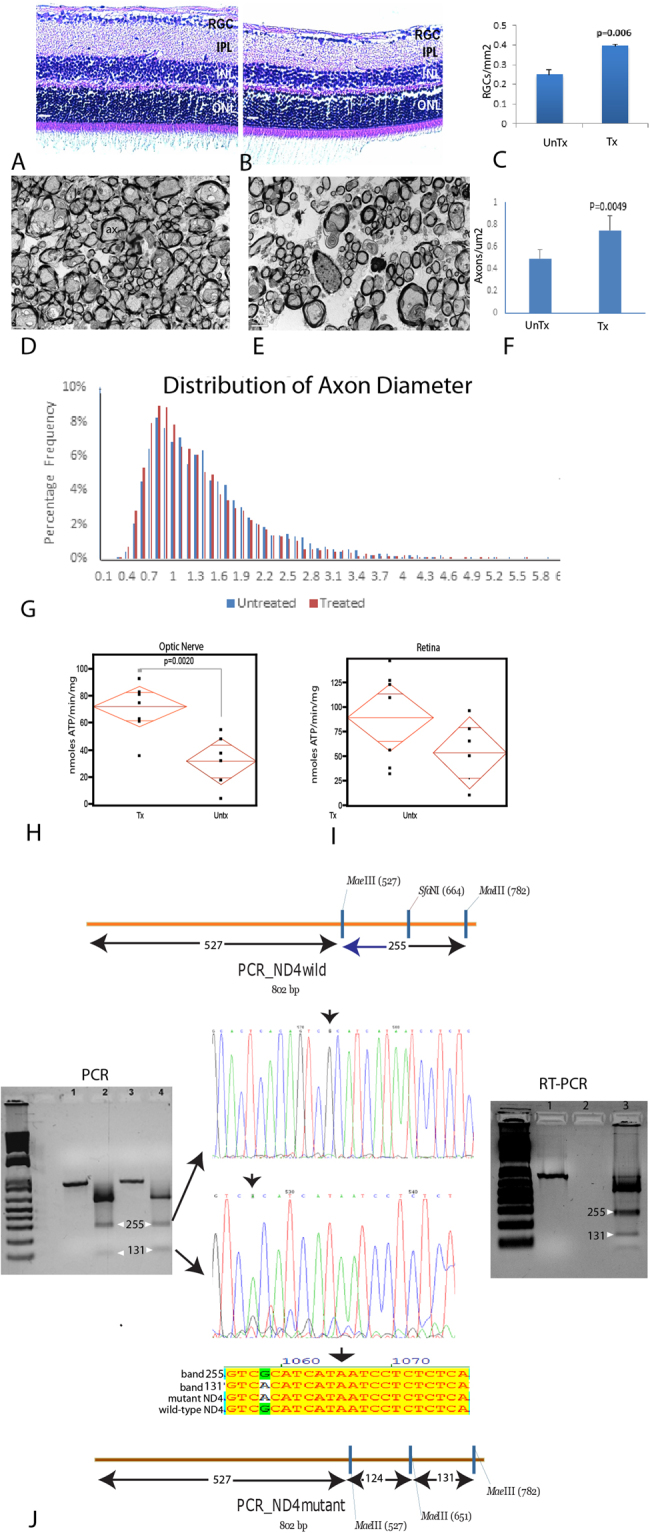
(**A**) Longitudinal retinal sections of an MTSAAV-*hND4* treated mouse and (**B**) an untreated mouse injected with MTSAAV-m*Cherry* at 15 months postinjection. Scale bars = 30 μm; (**C**) Bar plot of cell numbers in the RGC layer. (**D**) Transmission electron micrographs of the retrobulbar optic nerve of an MTSAAV-*hND4* treated mouse and (**E**) an MTSAAV-m*cherry* nerve. Ax = axon, arrows = electron dense debris in cystic spaces were axons are missing. Scale bars = 2 μm. (**F**) A bar plot showing axon counts in MTSAAV-*hND4* treated nerves and MTSAAV-m*cherry* controls. (**G**) A histogram of the percentage distribution of optic nerve axon diameters in control and treated mice (bin size, 0.1 μm). The distribution of axonal diameters in the MTSAAV-*hND4* treated mice shows preservation of small diameter axons with a shift to larger diameter axons in MTSAAV-m*cherry* injected controls. (**H**) Quantile scatterplots of ATP production in the optic nerves of MTSAAV-*hND4* treated and MTSAAV-m*cherry* controls. (**I**) Quantile scatterplots of ATP production in the retinas of MTSAAV-*hND4* treated and MTSAAV-m*Cherry* controls. (**J**) Agarose gel electrophoresis of an undigested 803 bp PCR amplification product spanning the G11778A point mutation (lanes 1 and 3) and MaeIII digested (lanes 2 and 4) PCR products from laser captured mtDNA of RGCs from MTSAAV-*hND4* treated affected mice. MaeIII cuts wild-type *ND4* into three bands of 527 bp, 255 bp and 21 bp, and cuts the mutant *ND4* into 4 bands of 527 bp, 124 bp, 131 bp and 21 bp (see illustrations). Sanger sequencing confirmed that the 255 bp band was wild-type human *ND4* and the 131 bp band was the mutant human *ND4*. An alignment shows the homology of the respective sequences between mutant and wild-type *ND4*. RT-PCR products from laser captured1RNA of RGCs from MTSAAV-*hND4* treated affected mice show MaeIII cuts wild-type *ND4* mRNA derived cDNAs into three bands of 527 bp, 255 bp and 21 bp, and cuts the mutant *ND4* cDNAs into 4 bands of 527 bp, 124 bp, 131 bp and 21 bp (see lane 3 RT-PCR agarose panel right, lane 1 is undigested cDNA).

To determine whether the increase in thickness of treated mice might represent swelling, as seen initially in LHON patients^13^, or true preservation of RGCs we next examined ocular histopathology. Light microscopy of the retina performed 15 months after intraocular injections, confirmed that *hND4* vector treated mice had more cells in the RGC layer and a thicker IPL (Fig. 2A) than the untreated mice (Fig. 2B). Quantitation of cells in the RGC layer revealed a 63% loss in cell numbers of control mice (5230 ± 494 cells/mm^2^; mean ± SD) relative to *hND4* vector treated mice (8286 ± 196 cells/mm^2^; mean ± SD), p = 0.006 (Fig. 2C).

Furthermore, ultrastructural analysis revealed that the optic nerves of wild-type *ND4* treated mice had numerous axons (Fig. 2D), while control animals had many cystic spaces and electron dense debris where axons were lost (Fig. 2E). Quantitative analysis revealed 51% more optic nerve axons in MTSAAV-*hND4* treated mitomice than in controls (p = 0.0049) (Fig. 2F). Analysis of the distribution of axon diameters revealed treated mitomice had more small axons (≤1 μm) than control eyes that had axons larger than 1 μm (Fig. 2G). These data suggest that delivered wild type *ND4* prevented the demise of small axons that are preferentially lost in human LHON^14^.

### Respiratory chain function in mutant ND4 mice

Because RGCs are adversely impacted by the mutant G11778A *ND4* and they are highly dependent on oxidative phosphorylation, we measured the rate of ATP synthesis by chemiluminescence with a modified luciferin-luciferase assay in freshly extracted digitonin-permeabilized optic nerve and retinal homogenates using malate and pyruvate that provide sources of NADH to complex I. We found that the rate of complex I-dependent ATP synthesis was significantly more robust in the optic nerves of wild-type *ND4* injected mice than in mock treated eyes of transgenic mice (Fig. 2H) (p = 0.002). Thus, the energy deficit in transgenic mutant *ND4* optic nerves was improved with wild-type *hND4* treatment. The reduction in ATP synthesis in untreated eyes was not statistically significant in the retina, perhaps because RGCs comprise a small fraction of the cells (<6%) (Fig. 2I).

### Wild-type human *ND4* DNA persisted in mice 15 months after injection

Next, we wished to determine the longevity of the MTS-AAV transferred DNA. To do so we tested for mutant and wild type human *ND4* DNA by laser microdissection of RGCs of MTSAAV-*hND4* treated mice followed by extraction of mitochondrial DNA and PCR amplification of an 803 bp fragment spanning the G11778A point mutation. We then digested the PCR products with MaeIII, which cuts wild-type human *ND4* into three bands of 527 bp, 255 bp and 21 bp, and cuts the mutated *ND4* into 4 bands of 527 bp, 124 bp, 131 bp and 21 bp (Fig. 2J). Sanger sequencing confirmed that the 255 bp band was wild-type human *ND4* and the 131 bp band was the mutant human *ND4*. Quantitative PCR of the ratio of human *ND4* to mouse *ND4* in RGCs was 31% but lower in the inner nuclear layer (2%) and outer nuclear layer (5%). As qPCR could not distinguish between mutant human and wild-type human *ND4* densitometry of the MaeIII digested bands revealed the ratio of wild-type to mutant human *ND4* DNA levels in RGC was 2.23. Thus transferred wild-type human *ND*4 persisted in RGCs for most of the lifespan of transgenic mutant *ND4* mitomice.

### Wild-type human *ND4* mRNA is transcribed in mice 15 months after injection

Lastly, we tested for transcription of the injected wild-type *ND4* as well as that of the transgenic mutant ND4 by laser microdissection of RGCs of MTSAAV-*hND4* treated mice followed by extraction of mitochondrial RNA and RT-PCR amplification of an 803 bp fragment spanning the G11778A point mutation. We then digested the RT-PCR products with MaeIII (see RT-PCR panel right in Fig. 2J), which cuts wild-type human *ND4* into three bands of 527 bp, 255 bp and 21 bp, and cuts the mutated *ND4* into 4 bands of 527 bp, 124 bp, 131 bp and 21 bp (Fig. 2J). The 255 bp band was wild-type human *ND4* cDNA and the 131 bp band was the mutant human *ND4* cDNA. Quantitative RT-PCR revealed that the ratio of human to mouse *ND4* cDNA was 245% in RGC’s but undetectable in the inner nuclear and outer nuclear layers. As qRT-PCR could not distinguish between mutant human and wild-type human *ND4* densitometry of the MaeIII digested bands revealed the ratio of wild-type to mutant human *ND4* DNA levels in RGC was 2.76. Thus, both wild-type and mutant human *ND4* were transcribed longterm 15 months after intravitreal injections suggesting it may likely do so in human LHON patients.

## Discussion

In our PERG experiments here we set the stimulus gratings to the largest size (0.05 c/deg = 2.77 logMAR^7–9^) and most contrast to detect severe visual deficits of our mice, as is also characteristic of human LHON, and to demonstrate reversal of them. The PERG amplitude of at least one eye of each transgenic mutant ND4 mouse randomized for treatment was at noise levels (2–3 uV), thus similar to blind G11778A LHON patients. The long lasting improvements in the PERG with treatment suggest these severe visual deficits were reversed for the most of the lifespan of our mice providing the preclinical data that similar improvements may be expected in LHON patients treated with this mitotargeted AAV containing wild-type human ND4 in future gene therapy trials.

Our work here complements the series of publications where we demonstrated that a mitochondrial-targeted AAV delivered the human wild-type or mutant *ND4* to mitochondria where the gene expresses episomally without integration into the endogenous host mtDNA^8,9,15^. Using this approach we were able to correct the deleterious effects of the G11778A LHON mutation in human mutant *ND4* cybrid cells. We then addressed the RGC and optic nerve degeneration of mice allotopically expressing the mutant human G11778A *ND4* in retinal ganglion cells^8^. We then generated a mouse that stably expresses episomally the human mutant G11778A ND4 by delivering the mitotargeted AAV in the zygote that transmitted the mutant human G11778A ND4 mutant episome maternally over many generations. Another group has independently developed an MTS-AAV showing import and expression of the transgene in mitochondria^16^. Taken together, our data show that the severe visual loss induced by a mitochondrial disease may be reversed for most of the lifespan of laboratory mice where it reduced loss of RGCs and their axons, improved oxidative phosphorylation with persistence of transferred ND4 DNA and transcription of ND4 mRNA. thus supporting mitotargeted AAV gene therapy as a long-term platform for treatment of human mitochondrial optic neuropathies such as LHON.

## Materials and Methods

### Plasmids and AAVs

scHSP-*hND4FLAG* + *mCherry* was constructed as previously described^8.9^. In brief, human *ND4FLAG* and mitochondrial-encoded *mCherry* were cloned into self-complementary (sc) AAV serotype 2 backbones under the control of the human mitochondrial heavy strand promoter (HSP), where *ND4FLAG* is followed by *mCherry* with a stop codon between two genes. Meanwhile, *mCherry* cloned in the same scAAV2 backbone was used as control (scHSP-*mCherry*). The plasmids were amplified and purified using the Qiagen endotoxin free maxiprep and then packaged with the VP2COX8GFP plus VP1, VP3 and helper plasmid PXX6 (3-fold excess) into recombinant virus: MTSAAV-*hND4* and MTSAAV-*mCherry*.

### Polymerase chain reaction (PCR) and reverse transcriptase–polymerase chain reaction (RT-PCR)

#### PCR

Mitochondria were isolated from micro-dissected cells from the retinal ganglion cell layer, inner nuclear layer and outer nuclear layer. DNA was extracted using DNeasy blood and tissue kit (Qiagen). For RNA extraction, mitochondrial were treated with 10 u DNase (Invitrogen) at 37 °C for 30 min followed by 75 °C 15 min. Then, RNA was extracted using the RNeasy mini kit (Qiagen) and used for reverse transcription (iScript cDNA Synthesis kit, Bio-Rad). PCR was performed with primers F11124: TCGAAACCACACTTATCC and R11926: TGATCAGGAGAACGTGG. PCR products were purified with QIAquick gel extraction kit (Qiagen) and digested with MaeIII. The interest fragments were recovered with the same kit listed above and sent for sequencing(Genewiz). Quantitative PCR assay was performed with oligonucleotides hND4F: 5′-CCTACTCATCGCACTAAT-3′ and hND4R 5′-TCATATTAAGTTATTGGCTCAG-3′: for human ND4; oligonucleotides mND4F: 5′-TTCATCCTTCTCTCCCTA-3′ and mND4R: 5′-ATTATTAGTATTGTTGCTCCTAT-3′ for mouse ND4 using Light cycle96 (Roche).

### Animals

All animal procedures were performed in accordance with the National Institutes of Health Guide for Care and Use of Laboratory Animals and the ARVO Statement for the use of Animals in Ophthalmic and Vision Research, with approval from the University of Miami Institutional Animal Care and Use Committee. For the intraocular injection of recombinant AAV, 24 mitomice of three months of age were sedated by inhalation with 1.5% to 2% isofluorane. A local anesthetic (proparacaine HCl) was applied topically to the cornea, and then a 32-gauge needle attached to a Hamilton syringe was inserted through the pars plana. Fourteen mice in the treated group were injected into both eyes with 1 µl of MTS-AAV carrying the WT human *ND4* (MTSAAV-*hND4*, titer = 4.4e^11^ vg/ml). Littermates (n = 10) in the control group were injected into both eyes with 1 µl of MTS-AAV carrying *mCherry* (MTSAAV-*mCherry*, titer = 3.08e^11^ vg/ml). PERGs were performed at baseline, and then again at 1, 3, and 13 months after intravitreal injections.

### PERG and OCT

In brief, mice were weighed and anesthesized with IP injections of a mixture of ketamine (80 mg/kg body weight) and xylazine (10 mg/kg body weight) and were restrained by using a bite bar and a nose holder that allowed unobstructed vision. The animals were kept at a constant body temperature of 37.6 °C with a feedback-controlled heating pad. In these conditions, the eyes of mice were wide open and in a stable position with undilated pupils pointing laterally and upward. The ERG electrode had a diameter of 0.25 mm, made of silver wire configured to a semicircular loop of 2-mm radius, was placed on the corneal surface by means of a micromanipulator and positioned in such a way as to encircle the pupil without limiting the field of view. Reference and ground electrodes were stainless-steel needles inserted under the skin of the scalp and tail, respectively. After setting the mice on the stage and before recording, a small drop of balanced saline was topically applied on the cornea to prevent drying. To detect the presence of absence of vision a visual stimulus of contrast-reversing bars (field area, 50° × 58°; mean luminance, 50 cd/m^2^; spatial frequency, 0.05 cycles/degree; contrast, 100%; and temporal frequency, 1 Hz) was aligned with the projection of the pupil at 20 cm distance. Eyes were not refracted for the viewing distance, because the mouse eye has a large depth of focus because of the pinhole pupil. Retinal signals were amplified (10,000-fold) and band-pass filtered (1–30 Hz). Three consecutive responses to 600 contrast reversals each were recorded. The responses were superimposed to check for consistency and then averaged. The PERG is a light-adapted response. To have a corresponding index of outer retinal function, a light-adapted flash ERG (FERG) was also recorded with non-dilated pupils in response to strobe flashes of 20 cd/m^2^/s superimposed on a steady background light of 12 cd/m^2^ and presented within a Ganzfeld bowl. Under these conditions, rod activity is largely suppressed while cone activity is minimally suppressed. Averaged PERGs and FERGs were automatically analyzed to evaluate the major positive and negative waves by Sigma Plot (Systat software Inc., San Jose, CA). Retinal images were visualized with *in vivo* spectral-domain (SD) OCT (Bioptigen, Inc.) and then were analyzed with semiautomated custom software written using MATLAB software (MathWorks, Inc.).

### Histology and Ultrastructure

At the appropriate time points rodents inoculated with the AAV vectors were overdosed with sodium pentobarbital. They were then perfused by cardiac puncture with PBS and then with fixative consisting of 4% paraformaldehyde in 0.1 M PBS buffer (pH 7.4). The eyes with attached optic nerves were dissected out and further processed by immersion fixation in 2.5% glutaraldehyde, postfixed in 1% osmium tetroxide, 0.1 M sodium cacodylate-HCl buffer (pH 7.4), 7% sucrose in the cold, and then dehydrated through an ethanol series to propylene oxide, infiltrated, and embedded in epoxy resin that was polymerized at 60 °C overnight. For immunocytochemistry, tissue specimens were postfixed in 5.0% acrolein, 0.1 M sodium cacodylate-HCl buffer (pH 7.4) and 7% sucrose and then dehydrated through an ethanol series and embedded in LR White (Ted Pella, Redding, CA) that was polymerized at 50 °C overnight. Examinations were made with a FEI CM10 transmission electron microscope, operating at 80 kV.

### Optic Nerve Diameters and Axons Counts

Fifteen months after intraocular injections, optic nerves of approximately 5 mm in length were dissected from 1 mm behind the globe to the optic chiasm. For axon counts, an average of 10 transmission electron micrographs were photographed by a masked observer at a magnification of 4600× for each optic nerve specimen. The diameter and number of axons were then manually counted. Axons were identified by a clear axoplasmic cytoskeleton or surrounding electron dense myelin sheath. A total of 2972 axons were counted for MTSAAV-m*cherry* controls and 4904 axons for MTSAAV-*hND4* treated mice.

### Statistical Analysis

Statistical analyses were performed using the Graphpad Prism software. Two groups were compared using two- tailed t tests, with P values of < 0.05 considered significant. Values were expressed as means ± standard deviation (SD).

## Electronic supplementary material

Supplementary Information
