# Supplementary material for: Longterm Reversal of Severe Visual Loss by Mitochondrial Gene Transfer in a Mouse Model of Leber Hereditary Optic Neuropathy
Source: Sci Rep. 2018 Apr 3;8:5587. doi: 10.1038/s41598-018-23836-y (PMC5882860; doi:10.1038/s41598-018-23836-y)
Supplement: Supplementary file 1 — Supplementary Information [file 41598_2018_23836_MOESM1_ESM.doc]

Longterm Reversal of Severe Visual Loss by Mitochondrial Gene Transfer in a Mouse Model of Leber Hereditary Optic Neuropathy

Hong Yu,^1^ Vittorio Porciatti,^1^ Alfred Lewin^2^, William Hauswirth^3^ and John Guy^1^


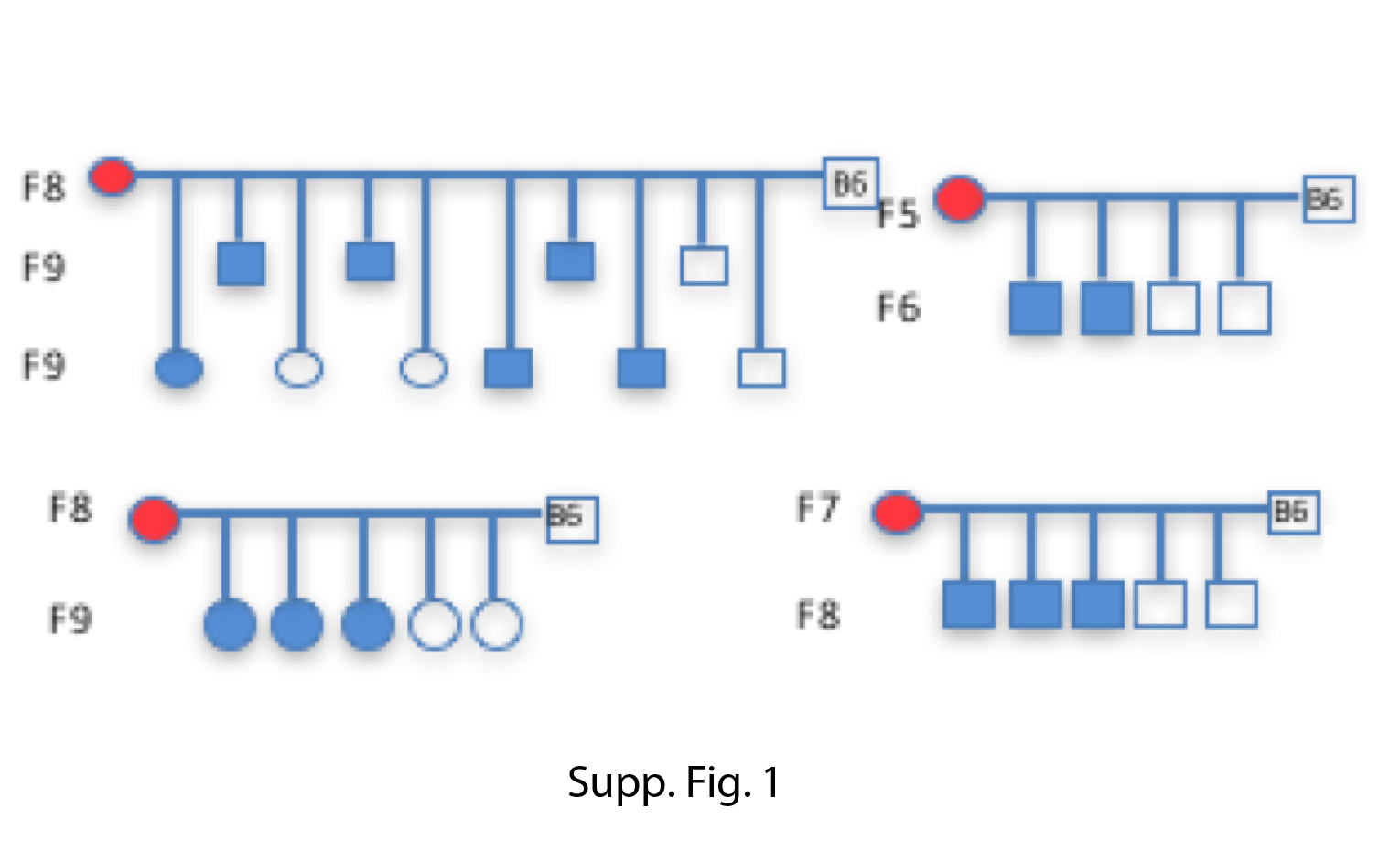


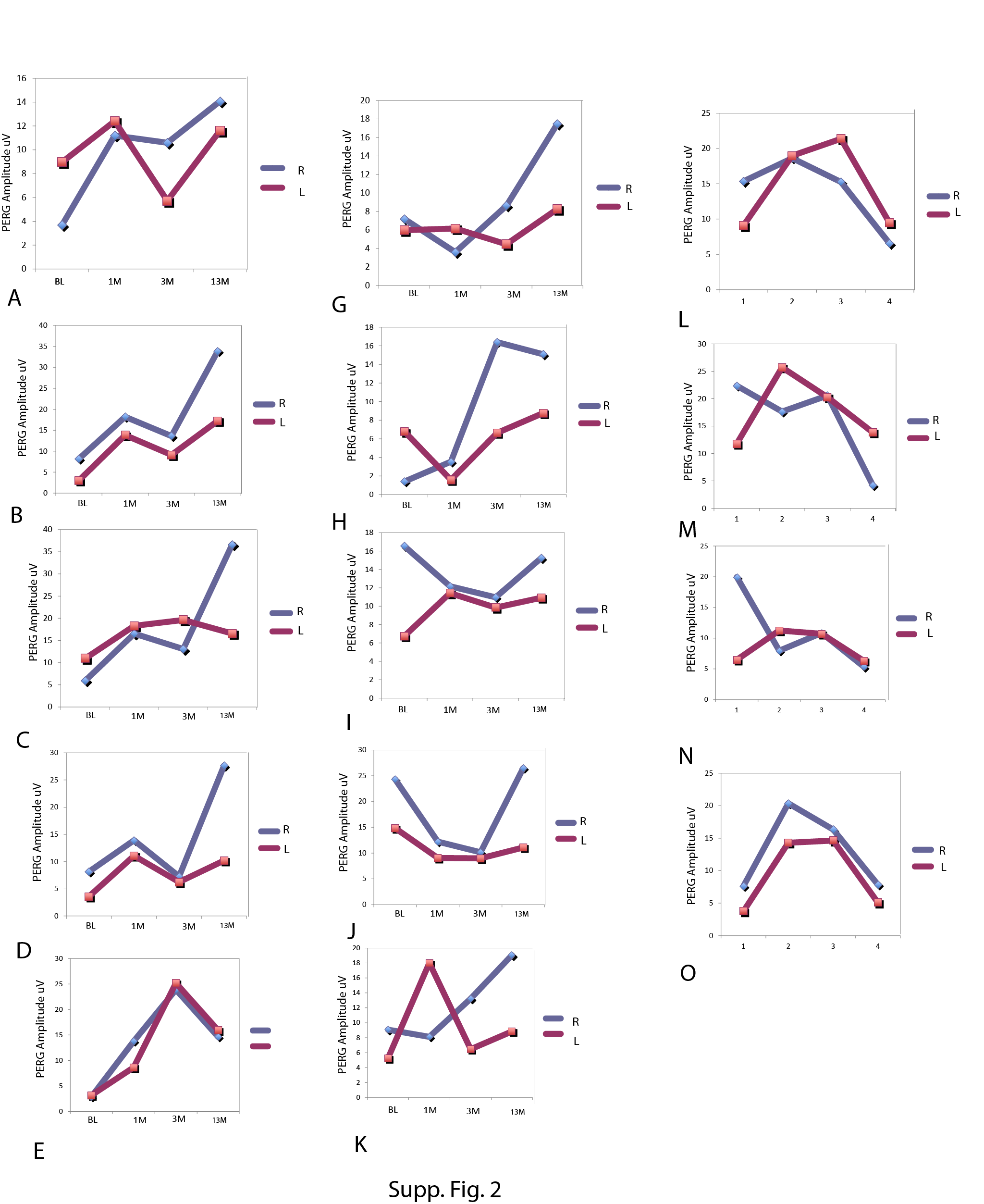


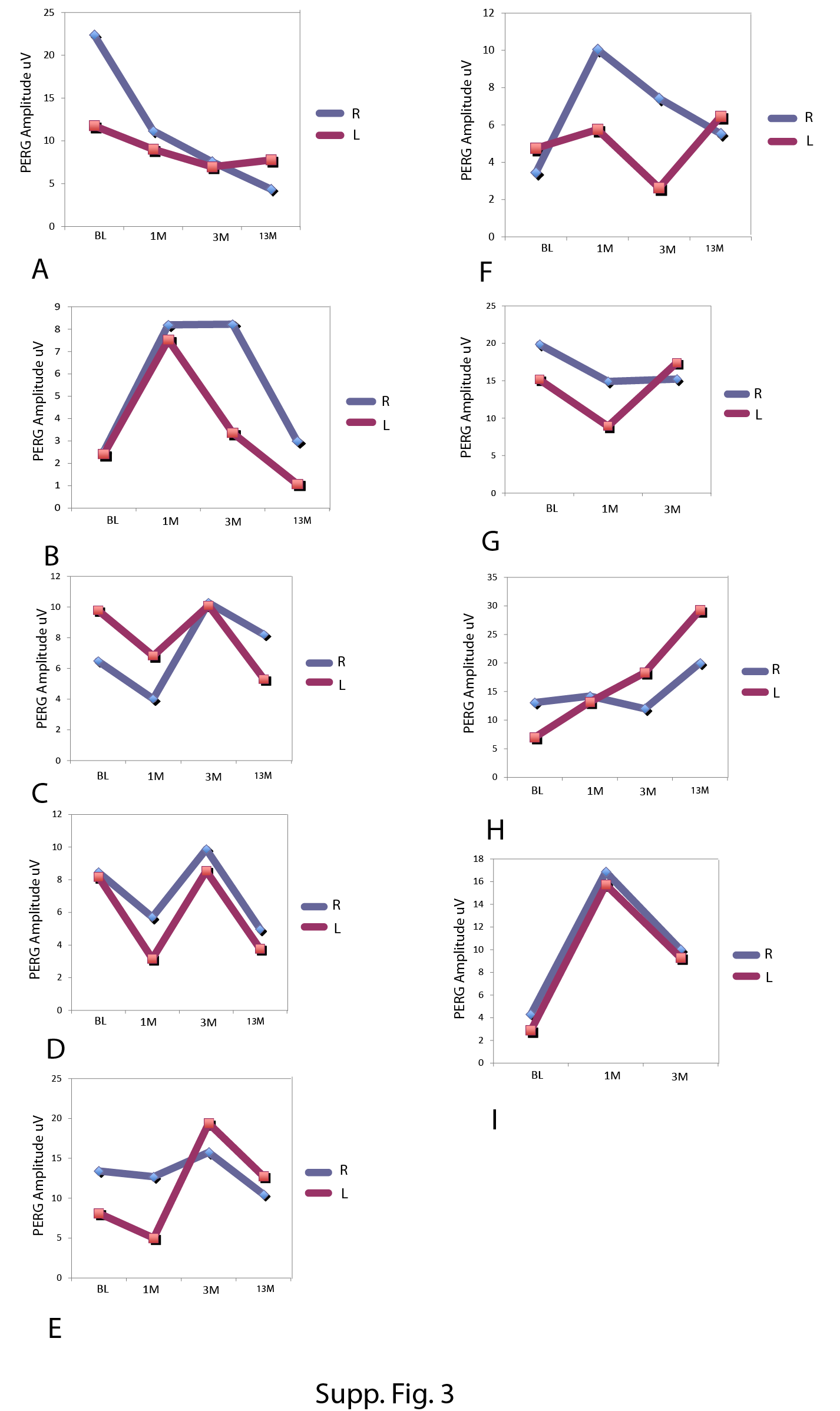


**Supp. Figure Legends**

**Supp. Fig. 1** - Genealogy tree of transgenic G11778A mitomice, filled circles (female) or squares (males)= injected with MTSAAV-hND4, open circles or squares = injected with MTS-AAV-mcherry. F5= fifth generation, F6 = sixth generation, F7 = seventh generation F8= eighth generation, F9 = ninth generation. Circles filled in red are selected female mitomice with high mCherry expression from each generation. Squares with B6 are C57BL/6J male mice.

**Supp. Fig. 2** – Plots of serial PERG amplitudes of right and left eyes of individual mice treated with hND4 vector.

**Supp. Fig. 3** – Plots of serial PERG amplitudes of right and left eyes of individual mice mock treated with mcherry vector.
